# The distance between the femoral nerve and anterior acetabulum is significantly shorter in hip osteoarthritis than in non-osteoarthritis hip

**DOI:** 10.1186/s12891-021-04295-5

**Published:** 2021-05-05

**Authors:** Kensuke Yoshino, Shigeo Hagiwara, Junichi Nakamura, Takuro Horikoshi, Hajime Yokota, Kenji Shimokawa, Koji Matsumoto, Yuki Shiko, Yohei Kawasaki, Seiji Ohtori

**Affiliations:** 1 Department of Orthopaedic Surgery, Graduate School of Medicine, Chiba University, 1-8-1 Inohana, Chuo-ku, Chiba, 260-8677 Japan; 2 Diagnostic Radiology and Radiation Oncology, Graduate School of Medicine, Chiba University, Chiba, Japan; 3 Biostatistics Section, Clinical Research Center, Chiba University Hospital, Chiba, Japan

**Keywords:** Femoral nerve, Acetabulum, Hip osteoarthritis, Iliopsoas muscle, Magnetic resonance imaging

## Abstract

**Background:**

The appropriate position of retractors to minimize the risk of femoral nerve palsy remains uncertain. The purpose of this imaging study was to evaluate the distance between the femoral nerve (FN) and anterior acetabulum (AA) in hip osteoarthritis (OA).

**Methods:**

Forty-one patients with unilateral hip OA underwent magnetic resonance imaging. Three measurement levels were defined and the minimum distance between the femoral nerve (FN) margin and anterior acetabulum (AA) rim was measured on axial T1-weighted images on the OA and normal sides at each level, with reference to an advanced neurography view. The cross-sectional area (CSA) of the iliopsoas muscle was also measured at each level bilaterally by three observers. Distances and CSAs were compared between the OA and normal side. Multiple regression analysis was performed to identify variables associated with the distance in OA.

**Results:**

The mean minimum FN to AA distances in OA were 19.4 mm at the top of the anterior inferior iliac spine (AIIS), 24.3 mm at the bottom of the AIIS, and 21.0 mm at the tip of the greater trochanter. These distances were significantly shorter than in normal hips at the top and bottom of the AIIS, with mean differences of 1.6 and 5.8 mm, respectively (*p* = 0.012, *p* < 0.001). CSAs of the iliopsoas in OA were significantly smaller at all levels (all *p* < 0.001), with reductions of 10.5 to 17.9%. The CSA of the iliopsoas at the bottom of the AIIS was associated with the FN to AA distance at the same level (*p* = 0.026). Interobserver reliabilities for measurements were very good to perfect (intraclass correlation coefficients 0.897 to 0.966).

**Conclusions:**

To minimize the risk of femoral nerve palsy, surgeons should consider the change of the femoral nerve to anterior acetabulum distance in osteoarthritic hip surgery.

## Introduction

Femoral nerve palsy following total hip arthroplasty (THA) is a serious neurological complication with a rate of 0.01–2.27% [1–3] that results in quadriceps femoris weakness with inability to extend the knee, and sensory disturbances that affect the anteromedial thigh or medial leg [4]. Previous study showed that an overall incidence of femoral nerve palsy was 0.21% in 17,350 primary THAs, with an incidence of 0.64% for the anterolateral approach, 0.40% for the direct anterior approach, 0.045% for the posterior approach, and 0.026% for the direct lateral approach [2]. These results indicated a higher risk of femoral nerve palsy in the anterolateral or direct anterior approach, and this has become a concern with recent increased use of this muscle sparing approach. However, the etiology is not well understood, with possible causes including compression from retractors or hematoma, traction and extension, direct injury, or thermal damage by electrocautery due to proximity of the femoral nerve (FN) to the anterior acetabulum (AA) [1–5].

Regarding anatomical features, a cadaveric study showed that the FN was closest to the AA rim at 90° from the anterior superior iliac spine, which correlated positively with the thickness of the iliopsoas muscle and the femoral length [5]. This indicated that placement of retractors at this point had a potential risk of femoral nerve injury during THA, especially in cases with a thin iliopsoas and short femoral length. However, this study investigated normal hip joints, rather than hips with osteoarthritis (OA), for which THA is indicated. Although there is little information regarding the distance in OA hips, preoperative muscle atrophy occurs in the lower extremity of patients with hip OA [6, 7], which suggests that the FN to AA distance, which is associated with iliopsoas thickness, may be shorter than in a normal hip. Therefore, there is a need to examine this distance and atrophy of the iliopsoas muscle in OA joints, with the goal of reducing the risk of nerve palsy in THA.

The objective of this study was to evaluate the distance between the FN and AA in OA hips, and to test the hypothesis that the FN becomes closer to the AA with an atrophic iliopsoas muscle in hip OA compared to a normal hip. In particular, we compared the distance between the FN and osseous AA using a novel method of magnetic resonance (MR) neurography in OA and normal hips, and evaluated the severity of iliopsoas muscle atrophy in the OA hip by measuring the cross-sectional area (CSA).

## Methods

This is the Level III diagnostic study, and was approved by our university institutional review board, and all subjects provided written informed consent.

### Participants

The subjects were 46 patients with unilateral hip OA who were awaiting THA and underwent magnetic resonance imaging (MRI) between July 2018 and November 2019. Radiological and physical examinations confirmed that the contralateral hip joint was normal without obvious dysplasia in all patients. Patients were excluded if they had previous hip trauma, previous surgery on the ipsilateral hip, a history of hip septic arthritis or neoplastic involvement, or diagnosis of neurological disease or inflammatory arthritis. Consequently, five patients were excluded (two acetabular osteotomies in childhood, one open reduction in infancy, one acetabular fracture, and one lymphoma invasion), leaving 41 patients who were included in the study. The sample size was evaluated using reported estimates for reliability studies using intraclass correlation coefficients (ICCs) [8, 9]. Patient demographics and clinical characteristics including Kellgren-Lawrence grade [10], Crowe classification [11], lateral center-edge angle, and modified Harris hip score (mHHS) were collected by multiple board-certified orthopedic surgeons and radiologists before MRI (Table 1).

**Table 1:** Patient demographics and clinical characteristics

Variables	OA (*N* = 41)	Normal (*N* = 41)	*P* Value
Age at imaging (years)	67.0 (43–82)	67.0 (43–82)	–
Sex (no. [%])			
Men	9 (22%)	9 (22%)	–
Women	32 (78%)	32 (78%)	–
Side (no. [%])			
Left	14 (34%)	27 (66%)	0.041*
Height (cm)	155.1 (137.1–171.8)	155.1 (137.1–171.8)	–
Weight (kg)	60.2 (35.8–133.0)	60.2 (35.8–133.0)	–
Body mass index (kg/m^2^)	24.8 (16.3–46.8)	24.8 (16.3–46.8)	–
Disease duration (months)	82.5 (10–456)	82.5 (10–456)	–
Flexion (°)	88.0 (60–120)	113.2 (90–130)	< 0.001^†^
Abduction (°)	20.9 (0–30)	30.0 (25–45)	< 0.001^†^
KL grade (no.[%])			
0–1	–	41 (100%)	< 0.001*
3	8 (20%)	–	
4	33 (80%)	–	
Crowe classification (no. [%])			
I	36 (88%)	41 (100%)	0.063*
II	5 (12%)	–	
LCEA (°)	22.5 (−24.9–45.4)	32.7 (21.2–50.8)	< 0.001^†^
mHHS (points)	40.8 (13.2–63.8)	75.6 (60.5–90.2)	< 0.001^†^

Values are given as a number with a percentage in parentheses or as a mean with the range in parentheses

*KL* Kellgren-Lawrence, *LCEA* Lateral center-edge angle, *mHHS* Modified Harris hip score

*Wilcoxon rank sum test

^†^paired t-test

### MRI procedure

All subjects underwent MRI with a 3.0-T system (Ingenia, Philips, Best, Netherlands) with a 16-channel standard coil. Coronal T1-weighted and 3D-NerveVIEW images were acquired along the anterior pelvic plane and reconstructed in axial planes to visualize the femoral nerve [12]. 3D-NerveVIEW is an advanced MR neurography sequence that consists of a three-dimensional (3D) T2-weighted image, a fat-saturated pulse and an improved motion sensitized driven equilibrium (iMSDE) pre-pulse for suppressing blood signals [13, 14]. MR parameters were as follows: coronal T1-weighted images (acquisition type = 3D, fat-saturation = mDIXON, repetition time = 4.4, echo time = 1.2/2.4, matrix = 208 × 208, field of view = 360 × 360 mm, % phase oversampling = 30%, slice thickness = 1.4 mm, slice gap 0.7 mm, voxel resolution = 1.44 × 1.44 × 1.4 mm, slice number 70); and coronal 3D-NerveVIEW (acquisition type = 3D, fat-saturation = short tau inversion recovery, iMSDE duration = 28 ms, repetition time = 1800, echo time = 88, inversion time = 270, matrix = 288 × 288, field of view = 300 × 300 mm, % phase oversampling = 30%, slice thickness = 3 mm, slice gap 0.5 mm, voxel resolution = 1.04 × 1.04 × 1.00 mm, slice number 210, acquisition time = 10 min 25 s).

### Measurement of the distance between the femoral nerve and acetabulum

**Fig. 1:**
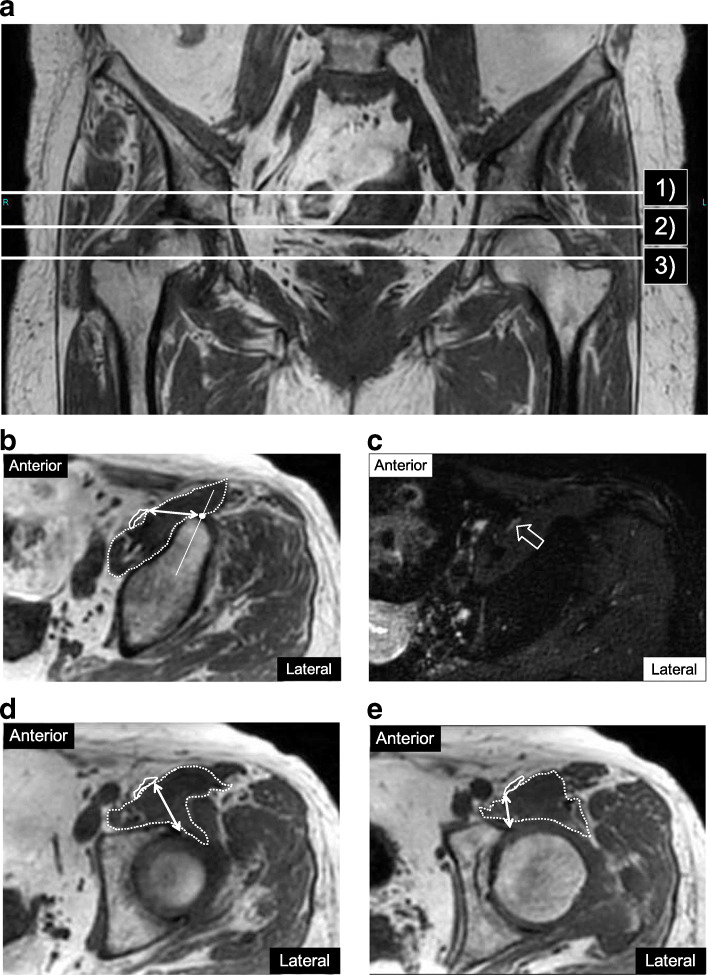
Magnetic resonance (MR) images showing measurements of the distance (white double-headed arrow) between the femoral nerve (FN) (solid line) and anterior acetabulum (AA), and the cross-sectional area (CSA) of the iliopsoas muscle (dotted line). **a** Measurement levels: (1) top of the anterior inferior iliac spine (AIIS), (2) bottom of the AIIS, (3) tip of the greater trochanter. **b** Axial T1-weighted image of the left acetabulum at level (1). The measurement point was defined as the intersection of the cortex and midline of the AIIS (white dot). The ambiguous FN was identified using the “nerve-sheath signal increased with inked rest-tissue rare imaging” (3D-NerveVIEW image. **c** Axial 3D-NerveVIEW image of the left acetabulum at the same level of B). The FN was clearly visualized in white (outline arrow). **d** Axial T1-weighted image of the left acetabulum at level (2). The measurement point on the acetabulum was defined as the head of the bony AA rim. The minimum FN to AA distance was measured from the lateral edge of the FN to the head of the acetabulum. **e** Axial T1-weighted image of the left acetabulum at level (3). The measurement point on the acetabulum was defined as the head of the bony AA rim. The minimum distance was measured from the medial edge of the FN to the head of the acetabulum

Three measurement levels were defined: 1) top of the anterior inferior iliac spine (AIIS); 2) bottom of the AIIS; and 3) tip of the greater trochanter, assumed to be near to the level of the center of the acetabulum (Fig. 1a) [15, 16]. The measurement point at the top of the AIIS was defined as the intersection of the cortex and the midline of the AIIS (Fig. 1b, c), and those at the bottom of the AIIS (Fig. 1d) and at the level of the tip of the greater trochanter (Fig. 1e) were both defined as the head of the bony AA rim. Computed tomography (CT) images taken around the same period for preoperative templating were used for bony contour identification if this was vague. The minimum distance from these points to the FN margin at each level was measured on axial T1-weighted images using a digital caliper tool in a Hope LifeMark picture archiving and communications system (Fujitsu, Tokyo, Japan) (Fig. 1b-e). Axial 3D-NerveVIEW images at each level were used to identify the FN (Fig. 1c). In cases with severe deformities of the anterosuperior acetabulum or femoral head, the measurement levels were defined as the plane perpendicular to the anterior pelvic plane, parallel to the tear drop line, and passing through each measurement point on the contralateral normal side. Each measurement was conducted independently by two board-certified radiologists (TH and HY) and one board-certified orthopedic surgeon (KY), and interobserver reliability was evaluated.

### Measurement of CSA of muscles

**Fig. 2:**
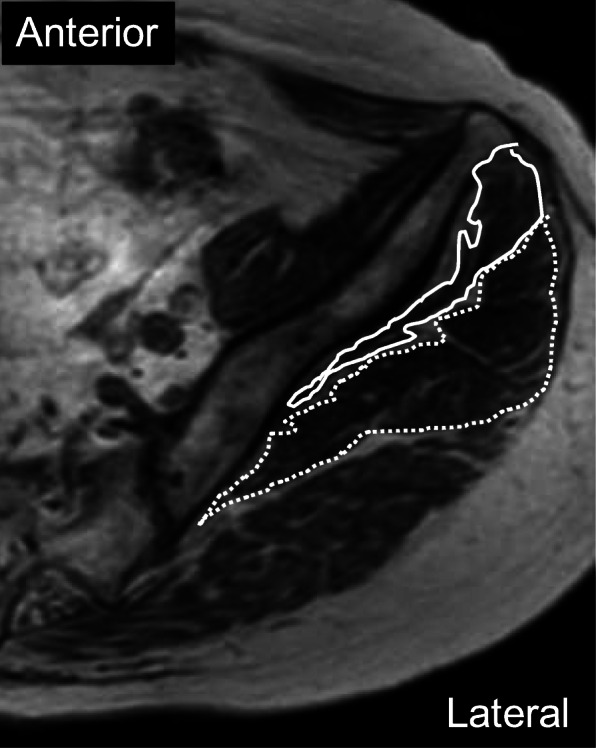
Axial T1-weighted image of the left pelvis at the plane through the bilateral anterior superior iliac spine. CSAs of the gluteus medius (dotted line) and gluteus minimus (solid line) muscles were measured by manually tracing fascia outlines

The CSA (mm^2^) of the iliopsoas muscle was determined at each measurement level, including the psoas major and minor, iliacus, and iliocapsularis muscles (Fig. 1b, d, e). CSAs of the gluteus medius and minimus muscles were also measured on the plane perpendicular to the anterior pelvic plane through the bilateral anterior superior iliac spine (Fig. 2). All CSA measurements were performed by manually tracing muscle fascia outlines [7] on axial T1-weighted images. Fatty infiltration of the muscles was excluded from the tracings. Each measurement was performed by two radiologists and one orthopedic surgeon, similarly to the distance measurements.

### Statistical analysis

Data are expressed as case numbers or means with a range. Statistical comparisons of demographic data between OA and normal hips were performed by Wilcoxon rank sum test for nominal valuables, and by paired t-test for continuous valuables. Distances between the FN and AA were compared by repeated measures analysis of variance (ANOVA) with a Tukey test. CSAs between two groups were compared by paired t-test.

Multiple linear regression analysis was performed to identify variables associated with the FN to AA distance at each measurement point in OA, with calculation of a regression coefficient with a confidence interval (CI) of 95% and a *p* value. This analysis used 22 variables: age, sex, height, weight, body mass index, disease duration, preoperative flexion and abduction, Kellgren-Lawrence grade, Crowe classification, total mHHS and scores for pain, limp, support, distance walked, sitting, public transportation, stairs, socks and shoes, and CSAs of the iliopsoas, gluteus medius and minimus on the OA side. Given the high correlation of the iliopsoas CSA among levels, only the CSA at the same level as the FN to AA distance was used. Variables that were significant in univariate regression analysis were used in the multiple linear regression analysis.

ICCs were calculated for interobserver reliability of the measurements of distances and CSAs, with an ICC value of 1 considered as perfect, > 0.80 very good, > 0.60 good, and > 0.40 moderate reliability [8]. A two-sided test with *p* < 0.05 was considered to be significant. All calculations were performed using SAS v.9.4 for Windows (SAS Institute, Cary, NC, USA).

## Results

OA hips were less frequent on the left side, and had lower flexion and abduction range of motion, a smaller lateral center-edge angle, and lower mHHS compared to normal hips (Table 1). There was no significant difference in the Crowe classification distribution between OA and normal hips.

**Fig. 3:**
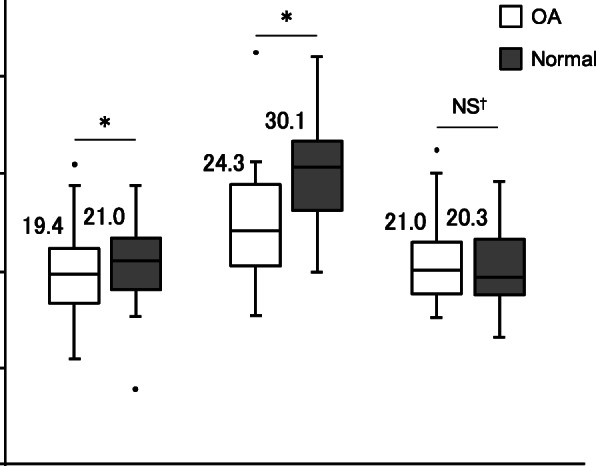
Box plots showing distances between the femoral nerve and anterior acetabulum at each measurement point. The numbers on boxes indicate means, the bottom and top of the boxes indicate 25th and 75th percentiles, and the horizontal line within indicates the median. The limits of the whiskers indicate values within the 1.5 interquartile range above and below the boxes; and dots indicate outliers. *p < 0.05, ^†^*p* = 0.270. AIIS, anterior inferior iliac spine; GT, greater trochanter; NS, not significant; OA, osteoarthritis

The mean minimum distances between the FN and AA on the OA side were 19.4 mm at the level of the top of the AIIS, 24.3 mm at the bottom of the AIIS, and 21.0 mm at the tip of the greater trochanter (Fig. 3). The distances were significantly shorter than in normal hips at the top (*p* = 0.012) and bottom of the AIIS (*p* < 0.001), with mean differences of 1.6 and 5.8 mm, respectively. In OA and normal hips, the FN to AA distance was significantly longer at the bottom of the AIIS than at the others two levels (both *p* < 0.001).

Considering sex, the mean minimum distances of females in OA were 18.8 mm at the level of the top of the AIIS, 23.6 mm at the bottom of the AIIS, and 20.0 mm at the tip of the greater trochanter. In males, the distances were 21.4 mm, 26.8 mm, and 24.7 mm, respectively. The distance in females was significantly shorter at the tip of the greater trochanter (*p* = 0.008), whereas there were no differences at the top (*p* = 0.106) and bottom of the AIIS (*p* = 0.264).

The CSA of the iliopsoas muscle in OA hips was significantly smaller than in normal hips at all levels (all *p* < 0.001), with reductions of 10.5 to 17.9% (Table 2). The CSA of the gluteus medius in OA hips was significantly smaller by 9.5% (*p* < 0.001), but the total CSA of the medius and minimus did not differ significantly between OA and normal hips (*p* = 0.166).

**Table 2:** Mean cross-sectional area (CSA) (mm^2^) of muscles and percentage differences between osteoarthritis (OA) and normal hips

	OA (range)	Normal (range)	% Difference (range)	*P* Value^†^
Iliopsoas				
Top of AIIS	740.5 (442.2–1127.2)	912.8 (592.6–1324.0)	−17.9 (−43.8 to 18.1)	< 0.001
Bottom of AIIS	624.2 (336.1–898.5)	760.7 (490.6–1079.2)	−16.7 (−54.7 to 11.7)	< 0.001
Tip of GT	503.8 (276.1–860.5)	575.3 (355.4–902.9)	−10.5 (−49.3 to 35.5)	< 0.001
Gluteus medius	2260.3 (1620.4–3252.9)	2539.1 (1463.7–3922.9)	−9.5 (−33.1 to 47.7)	< 0.001
Gluteus minimus	734.9 (263.2–1849.8)	566.7 (85.2–1236.6)	37.9 (−54.5 to 262.5)	0.001
Gluteus medius/minimus	2995.2 (1883.5–5102.6)	3105.8 (1548.9–5159.4)	−1.9 (−28.5 to 71.0)	0.166

*AIIS* Anterior inferior iliac spine, *GT* Greater trochanter

^†^paired t-test

At the top of the AIIS, the Crowe classification (*r* = 5.839, 95% CI 2.078 to 9.601, *p* = 0.003) and height (*r* = 0.167, 95% CI 0.022 to 0.313, *p* = 0.025) were associated with the FN to AA distance in multiple linear regression analysis. At the bottom of the AIIS, the CSA of the iliopsoas at the same level (*r* = 0.015, 95% CI 0.002 to 0.028, *p* = 0.026) was associated with this distance in single regression analysis, with no other relationships found. At the tip of the greater trochanter, no variable was associated with the FN to AA distance in multiple linear regression analysis. The sex was not associated with the FN to AA distance at all levels.

ICCs for interobserver reliabilities of distance and CSAs measurement are shown in Table 3. All these values indicated very good to perfect reliability.

**Table 3:** Interobserver intraclass correlation coefficients (ICCs) for measurement of the distance between the femoral nerve and anterior acetabulum and the cross-sectional areas (CSA) of muscles at each point

			ICC (95% CI)
Distance		Top of AIIS	0.961 (0.943–0.973)
		Bottom of AIIS	0.897 (0.848–0.931)
		Tip of GT	0.913 (0.873–0.941)
CSA	Iliopsoas	Top of AIIS	0.966 (0.936–0.981)
		Bottom of AIIS	0.952 (0.926–0.969)
		Tip of GT	0.910 (0.851–0.944)
	Gluteus medius		0.957 (0.893–0.979)
	Gluteus minimus		0.953 (0.923–0.970)

*AIIS* Anterior inferior iliac spine, *CI* Confidence interval, *GT* Greater trochanter

## Discussion

Femoral nerve palsy after THA is a serious neurological complication that leads to an inability to extend the knee and sensory disturbances in the thigh or leg. The etiology is poorly understood, but may involve damage by a retractor due to the closeness of the FN and AA. To minimize the risk of femoral nerve palsy, several reports suggest the suitable retractor position or insertion length [5, 15, 17], though these subjects were normal hip joints. The lack of evidence regarding the FN to AA distance in OA hips led to the current study, and there were three main findings. First, distances between the FN and AA in OA hips ranged from 19.4 to 24.3 mm from the level of the AIIS to the tip of the greater trochanter. This distance is closer at the level of the top and bottom of the AIIS compared to that in normal hips, and especially by a mean of 5.8 mm at the bottom of the AIIS. Second, the CSA of the iliopsoas muscle in OA decreased by 10.5 to 17.9%. Third, the CSA of the iliopsoas at the bottom of the AIIS was associated with the FN to AA distance at the same level. These results show that the hypothesis that the FN becomes closer to the AA with an atrophic iliopsoas muscle in hip OA is applicable at the level of the bottom of the AIIS, which had the largest difference in this distance between OA and normal hips.

The appropriate position of retractors to minimize the risk of femoral nerve palsy remains uncertain. The cause of femoral nerve palsy is suspected to be compression of the nerve through the iliopsoas muscle bulk by a retractor [18], or direct injury by a tip of a retractor [19]. Given these possible mechanisms, the FN to AA distance may be correlated with the risk for femoral nerve injury and it would be better to place a retractor where the FN is more distant. Previous reports suggested that retractors should be placed around the AIIS or superior to the 3 or 9 o’clock position of the AA to avoid damage to neurovascular structure [15, 17]. In the current study, the mean FN to AA distances in OA hips were 19.4 to 24.3 mm in these areas, 18.8 to 23.6 mm in females, and 21.4 to 26.8 mm in males. This finding suggests that a retractor placed around the AIIS and superior to the 3 or 9 o’clock position of the AA would be better within 18 mm in length to minimize the risk of femoral nerve palsy in OA hip surgery. There would be differences in the distances in sex and it might be safer in males considering the physical body size. Based on the results of multiple linear regression analysis, however, the FN to AA distances in OA hips would be shorter than in normal hips regardless of sex.

From a clinical viewpoint, the difference in FN to AA distance between OA and normal hips of 5.8 mm is a considerable concern. This difference was at the bottom of the AIIS, where the FN to AA distance was longest at a mean of 24.3 mm in the area superior to the 3 or 9 o’clock position. This may be the optimal point based only on the longer FN to AA distance. However, due to the increased FN to AA proximity compared to normal hips, care is needed even when placing the retractor in this area in OA hips.

Several previous studies have examined the FN to AA distance in normal hips, using CT, MRI or cadavers. At the 3 or 9 o’clock position, an almost similar level as the tip of the greater trochanter, the mean distance to the FN ranged from 18.0 to 23.6 mm [5, 15, 19–21]. At the anterosuperior acetabulum, which is approximately the level of the bottom of the AIIS, the mean distance was 26.5 to 33.2 mm [5, 15]. In OA hips, Wang et al. [21] found a mean distance at the 3 or 9 o’clock position of 20.6 mm, with no difference between pathologic and normal hips. Our results are consistent with these reports on both normal and OA hips, which indicates the reliability of the measurements in the present study with a coordinate system reconstructed in axial planes along the anterior pelvic plane and 3D-NerveVIEW imaging. This is supported by the interobserver ICC values that indicated good to perfect reliability.

Several studies of muscles around an OA hip have found decreases in CSA for the iliopsoas by a mean of 13.8% [6] and the gluteus medius by 15.0% [16], but no significant change for the total of the gluteus medius and minimus [6, 22]. Our results are similar to these findings; that is, the iliopsoas atrophied by 10.5 to 17.9%, but there was no change in the total area of the gluteus medius and minimus. The previous study in cadavers showed a positive correlation of the thickness of the iliopsoas muscle with the FN to AA distance [5]. Atrophy of the iliopsoas around the hip has received less attention than that of abductor muscles clinically. However, the present study suggests the importance of consideration of the iliopsoas muscle in hip surgery.

The Crowe classification and height were identified as variables associated with the FN to AA distance at the top of the AIIS in OA. In addition, the CSA of the iliopsoas at the bottom of the AIIS was related to this distance at the same level. The regression coefficients were all positive, which indicates that the FN to AA distance increases for a Crowe classification of group II, a tall patient, or a large CSA of the iliopsoas. A correlation of the distance with the thickness of the iliopsoas muscle and femoral length, which reflect physical body size [23], has been described previously [5]. The current results are consistent with this report. In the Crowe classification, the morphological changes in group II of the femoral head subluxated proximally and shifted laterally [11] may extend the FN to AA distance compared to group I.

This study has several limitations. First, the sample size was small and reliability was determined based on ICCs, rather than detectability of the difference in the FN to AA distance. However, our sample size could detect a difference between OA and normal hips of a mean of 1.6 mm, which is sufficient in clinical practice. More sample size would be needed when comparing the distances in sex. Second, the femoral nerve cannot be clearly identified distal to the level of the tip of the greater trochanter in MRI, which prevented evaluation of relationships at the distal level. Alternative assessing methods such as ultrasonography or other MR neurography sequences would help resolve this problem. Third, our subjects had primary and secondary OA, and morphological differences between these conditions might influence the distance. However, we showed that the contralateral hip was normal without obvious dysplasia radiologically, which indicates that OA hips were compared with normal hips, rather than painless dysplastic hips. Fourth, the imbalance of right and left side OA might have affected the results for distances or CSAs. However, there is no known association of bilateral differences in hip OA with clinical results or muscle volume around the joint.

## Conclusions

In conclusion, the femoral nerve is closer to the acetabulum with an atrophic iliopsoas muscle in hip OA at the level of the bottom of the AIIS. Iliopsoas atrophy reduces this distance by a mean of 5.8 mm. Recognition of this change is important for avoidance of femoral nerve injury in this area in osteoarthritic hip surgery.

## Data Availability

This work was performed at Chiba University Hospital, Chiba, Japan. The datasets generated and analysed during the current study are not publicly available due to limitations of ethical approval involving the patient data and anonymity but are available from the corresponding author on reasonable request.

## Abbreviations

THA: Total hip arthroplasty
FN: Femoral nerve
AA: Anterior acetabulum
OA: Osteoarthritis
MR: Magnetic resonance
CSA: Cross-sectional area
MRI: Magnetic resonance imaging
ICC: Intraclass correlation coefficient
mHHS: Modified Harris hip score
three-dimensional: 3D
iMSDE: Improved motion sensitized driven equilibrium
AIIS: Anterior inferior iliac spine
ANOVA: Analysis of variance
CI: Confidence interval
GT: Greater trochanter
